# Practical Notes and Formulæ

**Published:** 1879-07-20

**Authors:** 


### PRACTICAL NOTES AND FORMULÆ.

**Viburnum Prunifolium.**—Dr. J. C. McCoy, of Texas, writes: I have used this remedy in several cases of threatened abortion. I will mention one case. Mrs. S., living two miles from town, has miscarried four times in last three years, always at the end of third month of gestation; was called to see her about six months ago; found her having uterine hemorrhage and pains; gave her morphine hypodermically to relieve pain. I then gave her tincture viburnum in teaspoonful doses every three hours, until all symptoms of abortion subsided, then three times a day for two weeks. Had no more trouble with her until the 24th of last month, when I delivered her of a pair of boys weighing seven or eight pounds.

I have used it in menorrhagia with success.

I am now giving it to a case of sterility; will report when it is "proven."

I prepare it by getting the bark of the green root, dry it in the shade, macerate eight ounces in a pint of dilute alcohol fifteen days, and filter.

**Query.**—Dr. W. W. Carpenter writes from California: The secretion formed by the three pairs of salivary glands, to which are added the fluids furnished by the lingual and palatine glands, and the numerous follicular glands of the buccal mucous membrane, constitutes the saliva, the organic constituents of which are ptyaline and mucus, and the xanthroproteic reaction proves the presence of albumen also.

The food, uniting with that secretion, passes into the stomach, receives the gastric and mucous secretions of that organ when it becomes chyme. It then passes into the duodenum, where it receives the biliary and pancreatic secretions, making another change, and it becomes chyle. Now, all this is physiological. Is it physiological to reverse this order of nature and place chyle in the home of chyme—the stomach? If not, then is pancreatine a suitable remedy to administer by the stomach?

I ask for professional opinion.

[As pancreatine contained in the pancreatic juice has been shown to possess the property of emulsifying fatty matters passing from the stomach into the duodenum, we can see no reason why, in case where the natural secretion is deficient, pancreatine administered by the stomach may not, in some measure at least, substitute the deficiency by emulsifying the oily matters previous to their entrance into the duodenum. ED. W.]

**Pneumonia.**—Dr. R. E. Hutchins, of Miss., writes: I herewith enclose you my approval of Dr. Alban S. Payne's treatment of pneumonia. Think his article on this so fatal disease a valuable one, well worth the attention of every practitioner of medecine. I must confess that he has expressed my views on the treatment of pneumonia more fully and clearer than I could even dare to do. I will, however, say—hoping that some impetus may be given to Dr. Payne's views—that I

have treated pneumonia for the last two years, by using fly blister, opium, quinine, carb. ammonia, calomel and good chicken tea, with almost perfect success, and I am inclined to believe that these drugs are the *sine qua non* in the treatment of nearly every case of pneumonia.

**Hydrobromic Acid.**—The affinity of hydrobromic acid for bases is between that of hydrochloric and hydriotic acids. I have prescribed it most frequently in half-drachm doses well diluted.

1. ℞ Dilute hydrobromic acid.
   Syrup.... ....................................aa fl. ℥j.

Dose, a teaspoonful in water.

This is not unpleasant to the taste, and may be given to obtain the constitutional effect of bromine as usually administered in combination with a base. It also acts like other mineral acids in being tonic, refrigerant, solvent, alterative, etc., and is very useful in the bilious conditions, including fevers, where the morbid symptoms recede with the coating on the tongue. I use little else in remittent fever.

2. ℞ Sulphate of quinia........................ 15 to 80 grs.
   Dilute hydrobromic acid...................
   Syrup......................................aa fl. ℥j.

Dose, teaspoonful in water.

This is extremely bitter, and in this respect cannot be improved by other additions. Like other acidulous preparations it is incompatible with licorice. Bromine has the power of modifying, in a marked degree, the cerebral effects of quinine; hence the value of this combination, aside from the alterative and other properties of the acid. In all cases of intermittent fever, I continue an antiperiodic from ten to thirteen days after the paroxysm ceases, and for permanent and other satisfactory results, this combination has proved to be far superior in my hands to any other not containing acid.

3. ℞ Sulphate of cinchonia..................... 15 to 45 grs.
   Dilute hydrobromic acid ..................
   Syrup......................................aa fl. ℥j.

Dose, teaspoonful in water.

I can discover no difference in the effect of cinchonia and quinine, except that the latter is to be preferred as a stimulant. I prescribe cinchonia because of its cheapness.

4. ℞ Red iodide of mercury............................ 1 gr.
   Dilute hydrobromic acid.......................... fl. ℥j.
   Fl. ext. orange peel..............................
   Syrup..............................................aa ʒiv.

Dose, teaspoonful in water.

The iodide of mercury is decomposed, the bromide being formed with the elimination of the iodine in the form of hydriotic acid. Mercury may be given in this manner for a long time without producing ptyalism, the salt being rapidly excreted.

5. ℞ Tartar emetic.......................................... 2 grs.
   Denarcotized tincture of opium........................ fl. ʒi'.
   Dilute hydrobromic acid............................... fl. ℥j.
   Syrup to make.......................................... fl. ℥ij.

Dose, teaspoonful in water.

For acute bronchitis.

6. ℞ Syrup of bromide of iron............................ fl. ℨ iv.
Bromide of quinia...................................... 16 grs.
Dilute hydrobromic acid.............................. fl. ℥ j.
Syrup................................................... fl. ℨ iv.

Dose, teaspoonful in water.

The wide applicability of this tonic is readily suggested by its composition.

7. ℞ Subcarbonate of bismuth.............................. 80 grs.
Dilute hydrobromic acid................................ fl. ℥ j.

Dissolve and add

Saccharated pepsine...................................... 80 grs.
Syrup to make.............................................. fl. ℥ ij.

Filter. Dose, teaspoonful in water.

This is preferable to ammoniated citrate of bismuth with pepsine, because it is not only permanent in the bottle, but it is not precipitated in the stomach as is the citrate. Its indications are evident to the professional reader. To it may be added pancreatine, with or without the pepsine,—*New Preparations.*

**Simple Elixir.**—A good formula for this pleasant and convenient vehicle was given on page 219 of our last volume, and is as follows.

℞ Oil of orange.......................................... dr. j.
Oil cinnamon.............................................. gtts. x.
Oil anise................................................... gtts. v.
Oil bitter almond........................................ gtts. ij.
Alcohol..................................................... pints ij.

Dissolve the oil in the alcohol and add the tincture, and triturate carb. of magnesia oz. ij ; then add gradually 4½ pints of water ; filter or strain, and add 3 pounds loaf sugar.

This is a most pleasant menstruum in which to administer disagreeable drugs.

**Syphilitic Phthisis.**—In a case of syphilitic phthisis, reported by Dr. Ross to Richmond Academy of Medicine, the following prescription had a magical effect.

℞ Hydrarg. chlorid. corros............................ grs. i.

Potass. iodid.............................................. ℥ ss.

Ext. stilling. fl........................................... ℥ j.

Syr. sarsap. comp........................................ ℥ j.

Tinc. cinchon. comp.,.................................... ℥ iv

M. ft. sol. et sig. : One teaspoonful three times daily, after meals.

**Dyspepsia.**—

℞ Comp. tinct. gentian.

Fl. extract valerian..............................aa ℥ iv.

Tinct. nux vomica........................................ ℨ i.

Bi-carb. soda.............................................. ℨ iii.

Carbolic acid.............................................. gtts x. M

One teaspoonful after meals.

The carbolic acid is added to prevent acid fermentation in the stomach. It also serves to preserve the mixture.

**Intermittent fever.**—Dr. McCready, of Pennsylvania, writes : I have found very satisfactory results from the following receipts in fevers.

℞ Ferri phosphates.................................... ʒ ij.

Zinci sulph.......................................... ʒ ss.

Quini sulph.......................................... ʒ i. M

Three to twelve grs. may be given at intervals of one to four hours.

℞ Quini sulph......................................... ʒ i.

Piperin.............................................. ʒ ii

Salicin.............................................. ʒ iii.

Dose, three to twelve grs. every one to four hours.

**Hypodermic Injection of Ergotine.**—Either one of the two subjoined formulas can be used.

1. ℞ Ergotine (extract of ergot)...................... 38 grains.
Diluted alcohol...................................114 minims.
Glycerine.........................................114 " M

Each minim represents about one-sixth of a grain of ergotine. The dose is from five to twelve minims.

2. ℞ Ergotine.................................... 1 drachm.
Water.......................................... 1 fluid drachm.

Dissolve and filter. Dose, one to three minims or more.—*Drug. Circ. and Chem. Gazette.*

**Quinia with Opium.**—It is affirmed that opium added to quinine increases the efficiency of both articles, requiring a less quantity of each ingredient to give equal results; thus

Pulv. doveri...................................... grs. v.
Quinine........................................... grs. ii.

would be more effectual than three grains of quinine alone.

Sulph. morphia.................................... gr. ¼

Quinine........................................... gr. i equal in effect to ⅓ of a grain of the morphia alone.

Assafetida modifies opium. For instance the following combination is excellent to procure rest in nervous and hysterical females.

℞ Assafetida.
Pulv. doveri......................................aa gr. iv.

as a dose.

**Tinc. of Iodine in Cholera Infantum.**—This remedy, in doses of one drop every two to three hours, given in sweetened mint water, or mint toddy, has been found adapted to some cases. The following formula is suggested.

℞ Tinc. iodine..................................... gtts. x.

Aqua cinnamomi.................................... ℥ ij.

Carbolic acid..................................... gr. j. M

Dose, one teaspoonful every one to three hours.

